# Incidence and risk factors for intraoperative hypotension in neonates undergoing non-cardiac surgery: a retrospective cohort study

**DOI:** 10.1016/j.jped.2026.101592

**Published:** 2026-07-30

**Authors:** Jialian Zhao, Chunyi Jin, Guihang An, Mei Guo, Wenyuan Zhang, Yang Li, Dengming Lai, Jinfa Tou, Yue Jin

**Affiliations:** a Department of Anesthesiology, Children's Hospital, Zhejiang University School of Medicine, National Clinical Research Center for Children and Adolescents' Health and Diseases, Hangzhou, China; b Department of Neonatal Surgery, Children's Hospital, Zhejiang University School of Medicine, National Clinical Research Center for Children and Adolescents' Health and Diseases, Hangzhou, China; c Perioperative and Systems Medicine Laboratory, Children's Hospital, Zhejiang University School of Medicine, National Clinical Research Center for Children and Adolescents' Health and Diseases, Hangzhou, China

**Keywords:** Hypotension, Mean arterial pressure, Neonate, Non-cardiac surgery, Postoperative complications

## Abstract

**Objective:**

Intraoperative hypotension is associated with adverse postoperative outcomes, yet evidence in the neonatal population is limited. This study aimed to investigate the incidence, risk factors, and prognostic significance of intraoperative hypotension in this population.

**Methods:**

This single-center retrospective study included 1712 neonates undergoing non-cardiac surgery from 2020 to 2025 at a tertiary children’s hospital in China. Intraoperative hypotension was defined as a reduction in mean arterial pressure of more than 20% from the preoperative baseline and lasting for more than 5 min. Multivariate logistic regression identified independent risk factors.

**Results:**

The incidence of intraoperative hypotension was 59.8% (1024/1712). Independent risk factors included American Society of Anesthesiologists Physical Status Classification > Ⅲ (OR = 1.357, 95% CI: 1.079–1.706, *P* = 0.009), emergency surgery (OR = 1.323, 95% CI: 1.054–1.660, *P* = 0.016), surgery duration > 60 min (OR = 1.457, 95% CI: 1.162–1.826, *P* = 0.001), thoracic surgery (OR = 1.727, 95% CI: 1.053–2.831, *P* = 0.030), preoperative congenital heart disease (OR = 1.379, 95% CI: 1.117–1.702, *P* = 0.003), and preoperative acidosis (OR = 1.349, 95% CI: 1.036–1.757, *P* = 0.026). Higher gestational age (OR = 0.936, 95% CI: 0.891–0.983, *P* = 0.008) and neurosurgery (OR = 0.566, 95% CI: 0.385–0.832, *P* = 0.004) were protective factors. Intraoperative hypotension was associated with prolonged postoperative recovery and increased acute kidney injury and intraventricular hemorrhage (all *P* < 0.05).

**Conclusions:**

Intraoperative hypotension is highly prevalent in neonatal non-cardiac surgery and was associated with short-term outcomes. Identified risk and protective factors may guide perioperative hemodynamic optimization in this vulnerable population.

## Introduction

Intraoperative hypotension (IOH) is common in non-cardiac surgery and may directly induce ischemic injury to vital organs such as the heart, brain, and kidneys, potentially leading to multiorgan dysfunction [[1], [2], [3]]. Neonates are a vulnerable population with immature neurological and cardiovascular systems under general anesthesia, and the incidence of IOH can exceed 50% [4]. Anesthetic agents may suppress myocardial contractility, induce peripheral vasodilation, and reduce sympathetic tone, thereby lowering cardiac output and systemic vascular resistance, ultimately resulting in IOH [5,6]. Beyond compromising intraoperative organ perfusion, IOH substantially elevates the risks of postoperative acute kidney injury, brain injury, and necrotizing enterocolitis, and may adversely affect long-term neurodevelopmental outcomes [7]. Hence, evaluating the occurrence of intraoperative hypotension in neonates, identifying its risk factors, and verifying its impact on prognosis carry important clinical value and practical significance.

Existing studies on neonatal intraoperative hypotension are predominantly limited to small sample sizes [8,9]. Most investigations only focus on the incidence or individual risk factors, without a comprehensive evaluation of preoperative baseline profiles, intraoperative events, and postoperative clinical outcomes [10]. Moreover, there is currently no universally accepted definition of IOH in neonates. The absence of standardized diagnostic criteria for IOH hinders the comparability and generalizability of research results [7,11]. Thus, a large-sample study that can explore the prevalence, preoperative risk factors, and outcome implications of IOH in neonates undergoing general anesthesia is clinically meaningful for optimizing perioperative hemodynamic management and mitigating adverse postoperative outcomes.

## Methods

### Study design and patients

This single-center retrospective cohort study was conducted at the Children’s Hospital, Zhejiang University School of Medicine, National Clinical Research Center for Children and Adolescents' Health and Diseases, Hangzhou, China. Participants were neonates (Chronological age < 28 days) who underwent non-cardiac surgery between January 2020 and December 2025. The study protocol was approved by the Institutional Review Board (No. 2024-IRB-0379). Informed consent was waived due to the retrospective nature of the study and anonymization of all data.

All neonatal patients received intravenous or inhaled general anesthesia, and invasive or non-invasive blood pressure monitoring was performed throughout the surgical procedure. Baseline and preoperative characteristics, intraoperative variables, and postoperative outcome data were collected. Exclusion criteria were as: (1) receiving general anesthesia without tracheal intubation, (2) preoperative hypotension, (3) admitted with an existing tracheal tube, (4) Severe congenital heart disease (CHD), heart failure, or other conditions requiring vasoactive agents or inotropes for hemodynamic support (5) data loss. (Supplementary Fig. 1).

### Data collection

Patient demographics and perioperative parameters were collected from the clinical information system and anesthesia management system of Children’s Hospital, Zhejiang University School of Medicine, National Clinical Research Center for Children and Adolescents' Health and Diseases (Hangzhou, China). Baseline characteristics included gestational age at birth, chronological age, weight, sex, preterm birth, American Society of Anesthesiologists (ASA) physical status classification, surgical type, invasiveness of the procedure, and emergency surgery. Preoperative comorbidities including respiratory diseases, congenital heart disease, and anemia, as well as preoperative interventions such as oxygen supplementation and blood transfusion, and preoperative laboratory tests were also recorded.

Intraoperative parameters comprised surgery and anesthesia duration, intraoperative hypoxemia, hypothermia, blood transfusion, fluid input, and blood loss. Postoperative outcomes were collected, including length of hospital and Intensive Care Unit (ICU) stay, mechanical ventilation time, 30-day mortality, postoperative transfusion, unplanned reintubation, and reoperation. Other complications such as postoperative respiratory complications, acute kidney injury (AKI) and intraventricular hemorrhage (IVH) were also included. All data were extracted by two independent investigators; any discrepancies were resolved by a third researcher.

### Blood pressure measurement and hypotension definition

Blood pressure was monitored in all neonates, and values were electronically recorded throughout the entire surgical period, from the induction of anesthesia until departure from the operating room. Invasive blood pressure was measured every one minute, while non-invasive blood pressure was recorded every 3 to 5 min. Mean arterial pressure (MAP) was calculated as (systolic blood pressure + 2 × diastolic blood pressure)/3. Invasive measurements were prioritized when both invasive and non-invasive values were available. Intraoperative hypotension was defined as a reduction in MAP exceeding 20% from the preoperative baseline value for more than 5 consecutive minutes [9,10,12]. Preoperative baseline blood pressure was determined as the mean ward blood pressure value within 24 h preoperatively.

## Statistical analysis

Study subjects were stratified into an IOH group and a non-IOH group based on the occurrence of intraoperative hypotension. Continuous variables with normal distribution were reported as mean ± standard deviation, and compared by Student t-tests. Non-normally distributed continuous variables were described as median (interquartile range), with intergroup differences assessed via the Mann-Whitney U test. Categorical variables were presented as counts (percentages), and compared by the chi-square test or Fisher’s exact test. Variables with a P-value < 0.10 in univariate analysis were included in the multivariate logistic regression model (Forward, Likelihood Ratio). Results were expressed as odds ratios with corresponding 95% confidence intervals. A two-sided *P* < 0.05 was considered statistically significant. All statistical analyses were performed using IBM SPSS 26.0 software.

## Results

### Incidence and univariate analysis

A total of 1712 neonates receiving non-cardiac surgery with general anesthesia were enrolled, of whom 1024 developed IOH, with an incidence of 59.8% (Supplementary Fig. 1). Compared with the non-IOH group, neonates in the IOH group had significantly smaller gestational age, younger chronological age, and lower body weight, together with a higher proportion of preterm birth (all *P* < 0.05). The IOH group also showed a markedly higher proportion of ASA class > Ⅲ (57.8% vs 43.5%, *P* < 0.001) and emergency surgery (72.3% vs 67.0%, P = 0.020). A higher proportion of thoracic surgery (7.2% vs 3.5%) but a lower proportion of neurosurgery (5.8% vs 11.9%, *P* < 0.05) was found in the IOH group. Preoperative comorbidities were more prevalent in the IOH group, including respiratory disease, anemia, and especially CHD (70.2% vs 59.4%, *P* < 0.001). Notably, the IOH group exhibited a significantly higher rate of preoperative acidosis (24.1% vs 15.4%, *P* < 0.001). No significant difference was found in baseline mean arterial pressure and preoperative lactate level between the two groups. Details are shown in Table 1.

**Table 1:** Baseline and preoperative factors associated with intraoperative hypotension.

	Total *N* = 1712	IOH *N* = 1024	No IOH N = 688	*P*
**Gestational age (weeks)**	38 (37–39)	38 (36–39)	38 (37–39)	<0.001
**Chronological age (days)**	6 (3–15)	6 (2–14)	7 (3–16)	0.001
**Weight (gram)**	3100(2700–3500)	3000 (2600–3400)	3200 (2800–3600)	<0.001
**Sex (Male)**	957 (55.9)	570 (55.7)	387 (56.3)	0.811
**Preterm birth**	406 (23.7)	282 (27.5)	124 (18.0)	<0.001
**ASA classification**				<0.001
Ⅲ	821 (48.0)	433 (42.2)	389 (56.5)	
> Ⅲ	891 (52.0)	592 (57.8)	299 (43.5)	
**Emergency surgery**	1201 (70.2)	740 (72.3)	461 (67.0)	0.020
**Type of Surgery**				<0.001
General Surgery	1387 (81)	844 (82.4)	543 (78.9)	
Thoracic Surgery	98 (5.7)	74 (7.2)	24 (3.5)	
Otolaryngologic Surgery	25 (1.5)	17 (1.7)	8 (1.2)	
Urological Surgery	48 (2.8)	23 (2.2)	25 (3.6)	
Orthopedic Surgery	13 (0.8)	7 (0.7)	6 (0.9)	
Neurosurgery	141 (8.2)	59 (5.8)	82 (11.9)	
**Invasiveness of surgery**				0.191
Endoscopic surgery	576 (33.6)	332 (32.4)	244 (35.5)	
Open surgery	1136 (66.4)	692 (67.6)	444 (64.5)	
**Preoperative disease**				
Respiratory disease	193 (11.3)	138 (13.5)	55 (8.0)	<0.001
CHD	1128 (65.9)	719 (70.2)	409 (59.4)	<0.001
Anemia	213 (12.4)	142 (13.9)	71 (10.3)	0.029
**Preoperative therapy**				
Supplemental oxygen	225 (13.1)	160 (15.6)	65 (9.4)	<0.001
Transfusion	55 (3.2)	37 (3.6)	18 (2.6)	0.251
**Preoperative Lab**				
Acidosis	353 (20.6)	247 (24.1)	106 (15.4)	<0.001
Lactic acid	3.12 ± 1.96	3.11 ± 2.15	3.14 ± 1.64	0.773
Hematocrit	48.1(41.5–54.5)	47.9 (41.0–54.2)	48.4 (42.3–54.8)	0.160
**Preoperative Baseline MAP**	51 (49–53)	51 (49–53)	51 (49–53)	0.482

Data are presented as median (IQR) or number (%). ASA, American Society of Anesthesiologists; CHD, congenital heart disease; IOH, intraoperative hypotension; MAP, mean arterial pressure.

The surgery and anesthesia duration in the IOH group was 80 (50–114) and 135 (99–172) minutes, which were significantly longer than those in the non-IOH Group (*P* < 0.001). Regarding intraoperative adverse events, the IOH group showed significantly higher incidences of hypoxemia (12.5% vs 9.0%) and hypothermia (69.9% vs 62.6%) compared with the non-IOH group (all *P* < 0.05). The proportion of intraoperative blood transfusion and volume of fluid infusion were also higher in the IOH group (both *P* < 0.001). Regarding characteristics of IOH, the lowest MAP was significantly lower in the IOH group (33 mmHg [IQR: 29–36] vs. 42 mmHg [IQR: 39–45], *P* < 0.001), and the median duration of hypotension was longer (20 min [IQR: 11–36] vs. 0 min [IQR: 0–2], *P* < 0.001) (Table 2).

**Table 2:** Intraoperative characteristics of intraoperative hypotension.

	Total N = 1712	IOH N = 1024	No IOH N = 688	*P*
**Duration of surgery (min)**	75 (45–110)	80 (50–114)	65 (38–102)	<0.001
**Duration of anesthesia (min)**	130 (91–167)	135 (99–172)	118 (85–158)	<0.001
**Blood loss (mL)**	2 (1–5)	2 (1–5)	2 (1–5)	<0.001
**Hypoxemia**	190 (11.1)	128 (12.5)	62 (9.0)	0.024
**Hypothermia**	1147 (67.0)	716 (69.9)	431 (62.6)	0.002
**Transfusion**	186 (10.9)	138 (13.5)	48 (7.0)	<0.001
**Fluid input (mL)**	60 (45–100)	70 (50–104)	50 (40–89)	<0.001
**Characteristics of IOH**				
Lowest MAP	36 (31–41)	33 (29–36)	42 (39–45)	<0.001
Duration	9 (0–24)	20 (11–36)	0 (0–2)	<0.001

Data are presented as median (IQR) or number (%). IOH, intraoperative hypotension; MAP, mean arterial pressure.

### IOH and outcome

As presented in Table 3, neonates in the IOH group demonstrated significantly longer postoperative mechanical ventilation time (13 [[7], [8], [9], [10], [11], [12], [13], [14], [15], [16], [17], [18], [19], [20], [21], [22]] vs 10 [[6], [7], [8], [9], [10], [11], [12], [13], [14], [15], [16]] hours), longer ICU stay (2 [[1], [2], [3], [4], [5]] vs 1 [[1], [2]] days), and longer hospital stay (14 [[8], [9], [10], [11], [12], [13], [14], [15], [16], [17], [18], [19], [20], [21], [22], [23]] vs 10 [[7], [8], [9], [10], [11], [12], [13], [14], [15], [16], [17]] days) relative to the non-IOH group (all *P* < 0.001). The 30-day postoperative mortality was 2.2% in the IOH group and 1.0% in the non-IOH group, though this difference did not reach statistical significance (*P* = 0.057). Kaplan–Meier survival analysis also showed that neonates with IOH had a lower 30-day survival rate than those without IOH, although the difference did not reach statistical significance by the log-rank test (*P* = 0.187) (Supplementary Fig. 2).

**Table 3:** Postoperative outcome of intraoperative hypotension.

	Total N = 1712	IOH N = 1024	No IOH N = 688	*P*
**Postoperative mechanical ventilation time (hours)**	12 (7–20)	13 (7–22)	10 (6–16)	<0.001
**Length of postoperative ICU stay (days)**	1 (1–4)	2 (1–5)	1 (1–2)	<0.001
**Length of postoperative hospital stay (days)**	12 (7–21)	14 (8–23)	10 (7–17)	<0.001
**Mortality within postoperative 30 days**	30 (1.8)	23 (2.2)	7 (1.0)	0.057
**Postoperative respiratory complications**	552 (32.2)	360 (35.2)	192 (27.9)	0.002
**Postoperative AKI**	69 (4.0)	52 (5.1)	17 (2.5)	0.007
**Postoperative IVH**	165 (9.6)	130 (12.7)	35 (5.1)	<0.001
**Postoperative transfusion**	413 (24.1)	287 (28.0)	126 (18.3)	<0.001
**Unplanned reintubation**	13 (0.8)	10 (1.0)	3 (0.4)	0.207
**Unplanned reoperation**	32 (1.9)	21 (2.1)	11 (1.6)	0.498

Data are presented as median (IQR) or number (%). IOH, intraoperative hypotension; AKI, acute kidney injury; ICU, intensive care unit; IVH, intraventricular hemorrhage.

As for postoperative complications, the IOH group showed markedly higher rates of respiratory complications (35.2% vs 27.9%), acute kidney injury (5.1% vs 2.5%), and intraventricular hemorrhage (12.7% vs 5.6%) (all *P* < 0.05). Postoperative transfusion was also more frequently required in the IOH group (28.0% vs 18.3%, *P* < 0.001). No significant intergroup differences were detected in unplanned reintubation or unplanned reoperation (all *P* > 0.05) (Table 3).

### Multivariate analysis and risk factors

To further identify independent risk factors for intraoperative hypotension in neonates, variables with P values < 0.10 in univariate analysis were entered into a multivariate logistic regression model. As demonstrated in Table 4, independent protective factors against IOH included greater gestational age (OR = 0.936, 95%CI: 0.891–0.983, *P* = 0.008) and neurosurgery (OR = 0.566, 95%CI: 0.385–0.832, *P* = 0.004). Independent risk factors for IOH included ASA classification >Ⅲ (OR = 1.357, 95%CI: 1.079–1.706, *P* = 0.009), emergency surgery (OR = 1.323, 95%CI: 1.054– 1.660, *P* = 0.016), operative duration longer than 60 min (OR = 1.457, 95%CI: 1.162–1.826, *P* = 0.001), thoracic surgery (OR = 1.727, 95%CI: 1.053–2.831, *P* = 0.030), preoperative CHD (OR = 1.379, 95%CI: 1.117–1.702, *P* = 0.003), and preoperative acidosis (OR = 1.349, 95%CI: 1.036–1.757, *P* = 0.026).

**Table 4:** Risk factors for intraoperative hypotension.

	*P*	OR	OR 95% CI
**Gestational age (weeks)**	0.008	0.936	0.891–0.983
**ASA > III**	0.009	1.357	1.079–1.706
**Emergency surgery**	0.016	1.323	1.054–1.660
**Surgery time > 60min**	0.001	1.457	1.162–1.826
**Thoracic surgery**	0.030	1.727	1.053–2.831
**Neurosurgery**	0.004	0.566	0.385–0.832
**Preoperative CHD**	0.003	1.379	1.117–1.702
**Preoperative acidosis**	0.026	1.349	1.036–1.757

Variables with *P* < 0.10 in univariate analysis were entered into the multivariate logistic regression model, and the forward (likelihood ratio) method was used to identify independent risk factors. Data are presented as odds ratios with corresponding 95% confidence intervals. ASA, American Society of Anesthesiologists; CHD, congenital heart disease.

## Discussion

This large-sample retrospective cohort study including 1712 cases found a high incidence of intraoperative hypotension in neonates undergoing non-cardiac surgery with general anesthesia. IOH was significantly associated with prolonged postoperative recovery and an increased risk of complications. Higher ASA classification, emergency surgery, preoperative congenital heart disease, preoperative acidosis, surgery duration >60 min, and thoracic surgery were independent risk factors, while greater gestational age and neurosurgery were protective factors. Therefore, close monitoring and timely intervention of IOH are warranted in clinical practice, particularly for neonates with the aforementioned risk factors.

The definition of IOH in neonates remains controversial, mainly because blood pressure varies with gestational age, postnatal age, and birth weight [12]. In neonatal practice, hypotension is commonly defined using absolute or age-related thresholds, such as MAP below gestational age in weeks, MAP < 35 mmHg, or values below postnatal age-specific percentiles [4,11,13]. However, these criteria are primarily applied to circulatory assessment during the first 72 h or the first week after birth. Relative reductions from baseline blood pressure have also been used as diagnostic criteria [7,10]. Because the studied cohort included neonates from birth to 28 days of age, a relative reduction from each patient’s own preoperative baseline was considered more appropriate than a fixed absolute threshold. Therefore, the authors defined IOH as a ≥ 20% decrease in MAP from preoperative baseline. Future studies are needed to establish a standardized definition of neonatal IOH.

The incidence of intraoperative hypotension in neonates undergoing general anesthesia exceeded 50%, consistent with previous reports [5,9,14]. This can be attributed to the physiological immaturity of the neonatal cardiovascular system, limited autonomic regulation, and high sensitivity to anesthetic agents. Neonates have minimal myocardial contractile reserve and immature sympathetic control; anesthetics may further suppress myocardial function, vasodilate peripheral vessels, and reduce vascular tone, collectively decreasing cardiac output and systemic vascular resistance [15]. These factors form the pathophysiological basis for the high prevalence of IOH in this population. The present findings clinically confirm that neonates are at extremely high risk for IOH and warrant prioritized perioperative hemodynamic monitoring and management.

Univariate analysis showed that the IOH group had significantly smaller gestational age, lower postnatal age, lower body weight, more preterm birth, and higher ASA classification. These findings suggest that poorer preoperative baseline status is associated with higher risk of IOH, which aligns with previous findings [16,17]. Gestational age is considered a protective factor against IOH, which is consistent with the physiological characteristics of newborns, as preterm infants exhibit immature systemic circulatory regulation and baroreflex development [18]. A higher ASA classification suggests more severe underlying diseases, more comorbidities, and insufficient organ reserve capacity, making these neonates more susceptible to hemodynamic decompensation following anesthesia and surgical operation [16]. Compared with healthy newborns, those with CHD often have intracardiac shunts, ventricular hypertrophy, pulmonary hypertension, or cardiac dysfunction, resulting in significantly lower myocardial compliance and cardiac output regulation capacity that predispose them to greater intraoperative hypotension [19]. This study also identified preoperative acidosis as a risk factor for IOH, indicating that internal environment homeostasis and tissue perfusion status are closely associated with IOH. Therefore, for critically ill newborns with preoperative CHD, especially preterm infants, cardiac function should be optimized and acidosis corrected preoperatively, and more rigorous intraoperative monitoring of blood pressure and cardiac output should be implemented to prevent and promptly correct IOH.

In this study, emergency surgery, operation duration more than 60 min, and thoracic surgery were independent risk factors for IOH, whereas neurosurgery was a protective factor. Pediatric patients undergoing emergency surgery often present with preoperative fasting, volume depletion, and heightened stress, increasing hemodynamic lability [20]. Prolonged surgery increases anesthetic exposure, surgical stress, fluid fluctuations, and hypothermia risk, collectively predisposing to IOH [16]. Thoracic surgery may influence intrathoracic pressure and cardiopulmonary interactions, compromising circulatory stability [21]. Neurosurgery was protective, likely because most procedures in the studied center were minimally invasive and did not affect intrathoracic pressure, intra-abdominal pressure, or venous return, thereby minimizing interference with cardiac preload and afterload.

From a prognostic perspective, the IOH group experienced significantly longer postoperative mechanical ventilation duration, length of ICU stay and postoperative hospital stay. IOH was also associated with acute kidney injury, intraventricular hemorrhage, and respiratory complications, indicating its adverse impact on postoperative recovery [11,22]. The neonatal brain, kidneys, and intestines are extremely sensitive to ischemia. Intraoperative hypotension can lead to decreased perfusion pressure and microcirculatory dysfunction, which may precipitate acute organ injury and potentially affect long-term neurodevelopmental outcomes [23,24]. However, previous studies have reported inconsistent findings regarding the relationship between IOH and postoperative AKI or adverse neurodevelopment [25,26]. This discrepancy may be attributed to differences in study populations, IOH definitions, perioperative management, follow-up periods, and confounding adjustment strategies. Further multicenter prospective studies are needed to clarify this relationship.

This study has several limitations. First, as a single-center retrospective study, it is subject to potential selection and information biases. Second, measurement discrepancies between noninvasive and invasive blood pressure monitoring may have introduced additional error [27]. Third, long-term outcomes such as neurodevelopmental follow-up were not collected. Future multicenter prospective cohort studies are needed to establish individualized IOH warning thresholds for neonates and to develop risk prediction models integrating preoperative risk factors and intraoperative monitoring parameters, thereby enabling precise early warning and individualized management.

## Conclusion

This study confirms that intraoperative hypotension is a common intraoperative event and was associated with a series of postoperative complications and severely affects neonatal recovery, highlighting the importance of maintaining intraoperative hemodynamic stability. In neonates undergoing general anesthesia, independent risk factors for IOH include high American Society of Anesthesiologists physical status, emergency surgery, preoperative congenital heart disease, preoperative acidosis, longer surgery duration, and thoracic surgery. Higher gestational age and neurosurgery were identified as protective factors. Optimizing preoperative management and intraoperative monitoring, along with shortening operative time, may reduce the risk of IOH and potentially improve neonatal outcomes.

## Funding

This work was supported by the National Natural Science Foundation of China (grant numbers 82372159).

## CRediT authorship contribution statement

**Jialian Zhao:** Conceptualization, Data curation, Formal analysis, Methodology, Writing – original draft. **Chunyi Jin:** Data curation. **Guihang An:** Data curation. **Mei Guo:** Data curation. **Wenyuan Zhang:** Data curation, Formal analysis. **Yang Li:** Data curation. **Dengming Lai:** Formal analysis. **Jinfa Tou:** Conceptualization, Writing – review & editing. **Yue Jin:** Conceptualization, Methodology, Writing – review & editing.

## Conflicts of interest

The authors declare that they have no known competing financial interests or personal relationships that could have appeared to influence the work reported in this paper.

## Data Availability

The datasets used in the current study are available from the corresponding author upon reasonable request.

## Appendix Supplementary materials

Image, application 1
