# Supplementary material for: Incidence and risk factors for intraoperative hypotension in neonates undergoing non-cardiac surgery: a retrospective cohort study
Source: J Pediatr (Rio J). 2026 Jul 30;102(6):101592. doi: 10.1016/j.jped.2026.101592 (PMC13451805; doi:10.1016/j.jped.2026.101592)
Supplement: Supplementary file 1 [file mmc1.docx]

**JPED-D-26-00212_ Supplementary Material**

**Supplementary Figure 1** Study flow chart.

**Supplementary Figure 2** Kaplan–Meier survival analysis of intraoperative hypotension and mortality.
